# Effectiveness of a self-reporting yes/no survey for dementia screening—trial in Fukui, Japan

**DOI:** 10.3389/fnagi.2022.1029614

**Published:** 2023-01-04

**Authors:** Tadanori Hamano, Miwako Nagata, Rokuro Matsubara, Yukihiko Ikebata, Tatsuhiko Ito, Akihiro Ibe, Youshi Fujita, Yukinori Kusaka, Takahiro Tokunaga, Soichi Enomoto, Yoshinori Endo, Asako Ueno, Norimichi Shirafuji, Masamichi Ikawa, Kouji Hayashi, Osamu Yamamura, Yasunari Nakamoto

**Affiliations:** ^1^Second Department of Internal Medicine, Faculty of Medical Sciences, University of Fukui, Fukui, Japan; ^2^Department of Aging and Dementia (DAD), Faculty of Medical Sciences, University of Fukui, Fukui, Japan; ^3^Life Science Innovation Center, University of Fukui, Fukui, Japan; ^4^Department of Neurology, Nakamura Hospital, Echizen-city, Japan; ^5^Department of Psychiatry, Matsubara Hospital, Fukui, Japan; ^6^Department of Surgery, Ikebata Hospital, Echizen-city, Japan; ^7^Department of Psychiatry, Sukoyaka Silver Hospital, Fukui, Japan; ^8^Department of Internal Medicine, Ibe Hospital, Echizen-cho, Japan; ^9^Department of Neurology, Fujita Neurological Hospital, Fukui, Japan; ^10^Department of Environmental Health, University of Fukui, Fukui, Japan; ^11^Research Promotion Office, Shinseikai Toyama Hospital, Toyama, Japan

**Keywords:** dementia screening, self-reporting yes/no survey, Kihon checklist, epidemiology, Mini-Mental State Examination, long-term care insurance

## Abstract

**Background:** Early intervention for dementia patients is extremely important for the prevention of dementia. However, so far, it is not clear as to what kind of screening will be useful for the early detection of dementia.

**Objective:** We aimed to investigate the relationship between the results of a short self-reporting yes/no survey selected in Kihon Checklist, developed by the Japanese Ministry of Health, Labor and Welfare to identify older adults who are at risk of requiring support/care, and other original items developed by Dementia Prevention Team, Fukui, Japan, and Mini-Mental State Examination (MMSE) scores, and determine the diagnostic efficacy of the self-reporting yes/no survey.

**Methods:** Self-reporting yes/no surveys were conducted for 87,687 individuals aged ≥65 years, living in Fukui, Japan, and did not have Long-Term Care Insurance, Japan. According to the survey results, selected individuals were advised to visit a local hospital to be assessed with MMSE.

**Results:** Individuals who could not make a call by looking up phone numbers and manage their own deposits and savings at the bank or automatic teller machine (ATM) had an increased risk of low MMSE score (≤23; odds ratio: 2.74 [1.89–3.97]; 95% confidence interval: 2.12 [1.46–3.07]).

**Conclusions:** Self-reporting yes/no survey could effectively screen for dementia. Not being able to make a call by looking up phone numbers and not being able to manage their own deposits and savings at the bank or ATM are signs of dementia.

## Introduction

With an increase in the aging population, there is an increase in the prevalence of dementia worldwide, which is expected to triple by 2050 (GBD 2019 Dementia Forecasting Collaborators, 2022). Japan is the most aged society globally (Satake et al., 2016). Dementia greatly affects patients, their families, and society. Early interventions can effectively prevent dementia progression, including Alzheimer’s disease (AD; Ohara et al., 2017). However, dementia is underdiagnosed in several patients (Borson et al., 2007). Specifically, even in countries with advanced medical care systems, including European countries such as the UK, USA, and Canada, approximately 40%–70% of individuals with dementia are not formally diagnosed (Thyrian et al., 2012; Lang et al., 2017). Several approaches for improving dementia recognition include the “protective dementia case-finding scheme” initiated by the UK government (Le Couteur et al., 2013) and the “Annual Wellness Visit” for Medicare enrollees in the US, which includes detection of any cognitive impairment (Le Couteur et al., 2013).

Routine screening is not recommended since there is no evidence that it benefits patients with dementia (Chambers et al., 2017). Older patients avoid visiting family physicians (FPs) given their fear of being diagnosed with dementia. Additionally, patients who are screened positive often do not undergo an additional diagnostic assessment or receive specific treatment (Boustani et al., 2005). Eichler et al. (2015a) reported that subject measure impairment (SMI) may represent the first symptomatic manifestation of AD. However, the best practice for dementia screening remains unclear.

A long-term care insurance system was established in Japan in 2000 and has provided many services including appropriate social care and enriched many services including appropriated social care and care content for the disabled (Campbell and Ikegami, 2000). The number of older adults with mild rather than moderate-to-severe disabilities increased after the system was started. The long-term care insurance system was revised in 2006 to take preventative steps towards coping with this situation; projects were started to identify older adults who are vulnerable to disabilities and to intervene by providing nutritional education, guidance on oral care information, and exercise coaching. At that time, the Kihon Checklist (KCL) was developed by the Japanese Ministry of Health, Labor, and Welfare to identify older adults at risk of requiring care/support. The KCL is a simple self-reporting yes/no survey comprising 25 questions regarding instrumental and social activities of daily living, physical functions, nutritional status, oral function, cognitive function, and depressive mood.

**Figure 1:**
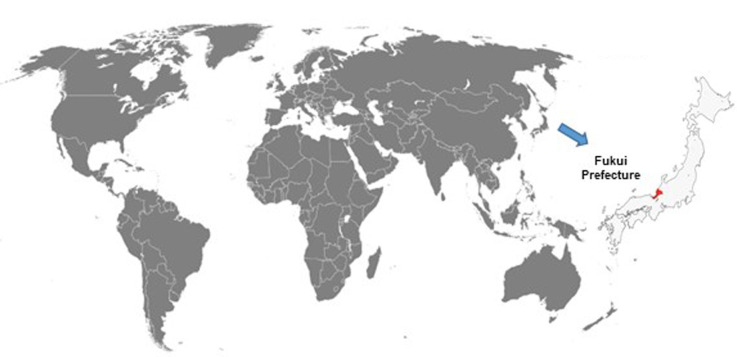
Location of Fukui prefecture, Japan.

In 2011, a dementia prevention campaign was started in Fukui Prefecture, Japan (Figure 1). As a tool for easy dementia screening, a self-reporting yes/no survey was selected by the Dementia Prevention Team in Fukui (DPTF; Authors TH, RM, MN, YI, TI, AI, and YF) based on this KCL, and original items selected by the DPTF. This study aimed to investigate the relationship between the results of this self-reporting yes/no survey and Mini-Mental state examination (MMSE) scores, as well as determine the diagnostic efficacy of this survey among patients who did not have Long-Term Care Insurance, Japan.

## Materials and Methods

### Study design and population

We performed a cross-sectional analysis of data derived from the ongoing Fukui-dementia prevention project. In 2014, the population of Fukui Prefecture was 789,633, with the number of individuals aged ≥65 years being 218,367. Table 1 lists the eligible individuals (aged >65 years and living in Fukui Prefecture, Japan at home without Long-Term Care Insurance, Japan) who were asked to complete the self-reporting yes/no survey. As these participants did not undergo Long-Term Care Insurance in Japan, they are usually expected to be able to live at home, have no major difficulties in daily living, and have no obvious impairment in cognitive function (Kitamura et al., 2022).

**Table 1:** List of self-reporting yes/no surveys to detect dementia.

**No**	**Questionnaire**	**Positive**	**Negative**
1.	Do you go outside by train, bus, or by driving a car by yourself?	No	Yes
2.	Do you go shopping for daily necessities by yourself?	No	Yes
3.	Do you manage your own deposits and savings at the bank or ATM?	No	Yes
4.	Have you recently noticed yourself asking the same thing repeatedly?	Yes	No
5.	Do you make a call by looking up phone numbers?	No	Yes
6.	Can you tell what‘s the date today?	No	Yes
7.	Do you lose your way?	Yes	No
8.	Have you lost interest in your favorite things?	Yes	No
9.	Do you often misplace things or forget to turn off the electricity?	Yes	No
10.	Have you experienced a recent loss of memory?	Yes	No
11.	Do you easily lose temper compared with before?	Yes	No

Patients who completed the self-reporting yes/no survey and sent it back *via* postal mail were screened for dementia.

### Procedure and instructions

The self-reporting yes/no survey items were as follows: #(1). Do you go outside by train, bus, or by a car driven by yourself? #(2). Do you go shopping for daily necessities by yourself? #(3). Do you manage your own deposits and savings at the bank or automatic teller machine (ATM)? #(4). Have you recently noticed yourself asking the same thing repeatedly? #(5). Do you make a call by looking up phone numbers? #(6). Can you tell what is the date today? #(7). Do you lose your way? #(8). Have you lost interest in your favorite things? #(9). Do you often misplace things or forget to turn off the electricity? #(10). Have you experienced a recent loss of memory? #(11). Do you easily lose temper compared to before? (Table 1). These self-reporting yes/no survey items, #1, #2, #3, #4, #5, and #6 were based on the KCL developed by the Japanese Ministry of Health, Labor, and Welfare to identify older adults at risk of requiring care/support (Satake et al., 2016). KCL was originally composed of 25 items, and six items were selected by DPTF. The DPTF determined #7, #8, #9, #10, and #11 as expert opinions. The basic checklist identifies high-risk older adults who will need nursing care in the near future (those eligible for secondary prevention projects).

Individuals with at least three positive items for questions 1–6 or at least one positive item for questions 7–11, respectively, were advised to visit their FPs.

The selected individuals underwent assessments of cognitive status using the Japanese version of MMSE (Ideno et al., 2012). The total MMSE score was categorized as follows: scores 28–30, 24–27, 19–23, 10–18, and <9 indicated no, minimal, mild, moderate, and severe cognitive impairment, respectively.

### Mini-Mental State Examination

The original version of the MMSE was developed by Folstein et al. (1975), and it remains a widely used screening tool for dementia. The Japanese version of the MMSE has been translated from the original version and validated by Ideno et al. (2012).

### Study sample

The self-reporting yes/no surveys were sent *via* postal mail to individuals who were aged ≥65 years in April 2014.

### Statistical analysis

We summarized variables that describe the sampling description statistics. We performed multiple logistic regression analyses to examine the relationship between the self-reporting yes/no survey results and the MMSE score, with adjustments for age and sex. We actually divided MMSE into 23 points or less and 24 points or more, and derived these values by binary logistic analysis. The predictive value and specificity for positive responses were obtained from the receiver operating characteristic (ROC) derived from the logistic regression (LR) model. All statistical analyses were performed using IBM SPSS Statistics (version 27, IBM Corp, Armonk, NY, USA). Statistical significance was set at *p* < 0.05.

## Results

### Study sample

Self-reporting yes/no surveys for dementia screening were conducted for a total of 87,867 individuals, and 51,043 (58.1%) individuals sent them back. Among them, 8,803 (17.2%) individuals were advised to visit their FPs to check the possibility of dementia.

**Figure 2:**
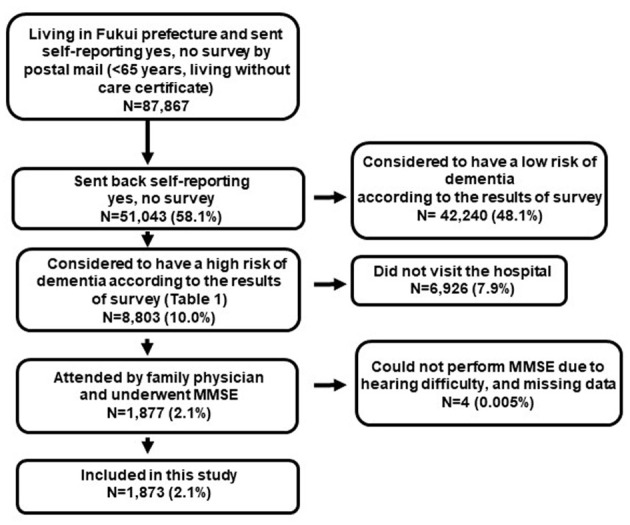
Flow chart detailing the derivation of the study sample.

A total of 1,877 (21.3%) individuals (805 men, 1,072 women, mean age: 76.8 ± 6.4 years) visited the local hospital; however, two individuals could not undergo the MMSE due to hearing problems, and 2 excluded due to missing data. Accordingly, this study included 1,873 patients (803 men, 1,070 women, mean age: 76.8 ± 6.4; Figure 2). The mean MMSE score was 27.0 ± 3.3. Table 2 describes the number of participants in each group. According to the age groups, the mean MMSE scores were as follows: 65–65 years, 28.5 ± 2.1; 70–74 years, 27.7 ± 2.8; 75–79 years, 27.0 ± 3.0; 80–84 years, 26.3 ± 3.6; and ≥85 years, 24.8 ± 3.9 (Table 2). Further, there were 995 (53.1%), 652 (34.8%), 180 (9.6%), 43 (2.3%), and 3 (0.2%) participants with MMSE scores of 28–30, 24–27, 19–23, 10–18, and ≤9, respectively (Table 3). The positive responses of the self-reporting yes/no survey were as follows: Do you misplace things or forget to turn off the electricity? Yes, 77.6%; Can you tell what is the date today? No, 66.5%; Have you experienced a recent loss of memory? Yes, 58.3%; Have you lost interest in your favorite things? Yes, 47.0% (Table 4).

**Table 2:** The population and mean MMSE scores of each age group.

**Age**	**Number of participants (%)**	**Mean MMSE score**
65–69	284 (15.2)	28.5 ± 2.1
70–74	379 (20.2)	27.7 ± 2.8
75–79	570 (30.4)	27.0 ± 3.0
80–84	443 (23.7)	26.3 ± 3.6
≥85	197 (10.5)	24.8 ± 3.9

**Table 3:** Distribution of participants’ MMSE scores.

**MMSE**	**Number of participants (%)**
28–30	995 (53.1)
24–27	652 (34.8)
19–23	180 (9.6)
10–18	43 (2.3)
≥9	3 (0.2)

**Table 4:** Positivity of each questionnaire to detect dementia in the participants.

**No.**	**Questionnaire**	**Answer(+)**	**Positivity(%)**
1.	Do you go outside by train, bus, or by driving a car by yourself?	No	18.2
2.	Do you go shopping for daily necessities by yourself?	No	13.2
3.	Do you manage your own deposits and savings at the bank or ATM?	No	15.4
4.	Have you recently noticed yourself asking the same thing repeatedly?	Yes	34.9
5.	Do you make a call by looking up phone numbers?	No	13.4
6.	Can you tell what’s the date today?	No	66.5
7.	Do you lose your way?	Yes	15.0
8.	Have you lost interest in your favorite things?	Yes	47.0
9.	Do you often misplace things or forget to turn off the electricity?	Yes	77.6
10.	Have you experienced a recent loss of memory?	Yes	58.3
11.	Do you easily lose temper compared with before?	Yes	26.6

Multivariate analysis revealed that individuals who do not make a call by looking up phone numbers (*P* < 0.0001, odds ratio (OR) 2.74, 95% CI 1.89–3.97), could not manage their own deposits and savings at the bank or ATM (*P* < 0.0001, OR 2.12, 95% CI 1.46–3.07), cannot tell what day today is (*P* < 0.0001, OR 2.03, 95% CI 1.40–2.96), and repeatedly asked the same things (*P* < 0.0001, OR 1.98, 95% CI 1.45–2.70) had an increased risk of low MMSE scores (≤23; Table 5).

**Table 5:** Relationship of the self-reporting yes, no survey results and the risk of dementia (MMSE <23).

**No.**		**Answer**	**Odds ratio**	**95% CI**	***P* value**
5.	Do you make a call by looking up phone numbers?	No	2.74	1.89, 3.97	<0.0001
3.	Do you manage your own deposits and savings at the bank or ATM?	No	2.12	1.46, 3.07	<0.0001
6.	Can you tell what’s the date today?	No	2.03	1.40, 2.96	<0.0001
4.	Have you recently noticed yourself asking the same thing repeatedly?	Yes	1.98	1.45, 2.70	<0.0001
1.	Do you go outside by train, bus, or by driving car by yourself?	No	1.78	1.23, 2.56	0.002

The predictive value and specificity for positive responses obtained from the ROC derived from the LR model to each self-reporting yes/no survey items were as follows: not make a call by looking up phone numbers = positive (31.6%/89.6%), could not manage their own deposits and savings at the bank or ATM = positive (38.8%/87.8%), and not telling what day today = positive (81.3%/35.5%), repeatedly asking the same things = positive (56.3%/67.9%), and inability to go outside by themselves = positive (41.5%/85.1%).

## Discussion

### Main findings and interpretation

This study examined the effectiveness of a self-reporting yes/no survey in screening early-stage dementia. Our findings demonstrated that individuals who cannot make a call by looking up phone numbers, cannot manage their own deposits and savings at the bank or ATM, cannot tell what day today is, repeatedly ask the same things have an increased risk of low MMSE scores and should visit the physician to rule out the possibility of dementia. The predictive value and specificity for positive responses to not make a call by looking up phone numbers and inability to manage their own deposits and savings at the bank or ATM were (31.6%/89.6%) and 38.8%/87.8%), respectively.

These self-reporting yes/no surveys may allow for early screening. Early dementia detection allows adequate access to information regarding social support and evidence-based treatment for patients with dementia (Prince et al., 2013). Donepezil administration, which ameliorates the progression of hippocampal atrophy, has been proposed as an early intervention for patients with dementia, including those with mild cognitive impairment (Woods et al., 2012). Furthermore, aducanumab, an amyloid-beta protein oligomer antibody, was approved by the Food and Drug Administration in June 2021 (Dunn et al., 2021). Therefore, this screening tool could greatly improve the well-being of the public.

### Comparison with literature

Arabi et al. (2016) investigated the reliability and construct validity of the Early Dementia Questionnaire (EDQ) on 200 older individuals. EDQ comprises 20 items, including requiring a checklist for memory support, difficulty following a conversation, and difficulty in taking care of personal hygiene. They reported that the sensitivity and specificity of the EDQ were 71.2% and 59.5%, respectively. Other questionnaires for dementia screening include the Informant Questionnaire on Cognitive Decline and the quick dementia rating system. However, there has been limited research in primary care settings (Harrison et al., 2014; Galvin, 2015), and future studies should consider comparing these questionnaires.

Thyrian et al. (2016) reported that 89.9% of general practitioners agreed that implementing a brief cognitive screen test is helpful. We attempted to identify individuals with dementia using the questionnaire. Our findings showed that individuals who do not go outside by bus or by driving themselves and those who do not go shopping by themselves have an increased risk of a low MMSE score (≤23).

The activity of daily living is an important factor for predicting cognitive dysfunction. Previous studies have reported the relationship between the scores of the activities of daily living questionnaire (ADLQ) score and the Montreal Cognitive Assessment (MoCA). Specifically, there is a negative correlation between the MoCA score and the level of functional impairment (Durant et al., 2016). Additionally, the ADLQ score was found to have a significant negative and positive correlation with the scores of the MMSE and Clinical Dementia Rating Scale, respectively (Johnson et al., 2004).

Eichler et al. (2015b) reported that screening for dementia could improve dementia recognition in primary care settings. Among 146 individuals without a formal dementia diagnosis, 49% received a formal diagnosis after a positive screening outcome (69% had unspecified dementia).

Satake et al. (2016) reported that the KCL is a useful tool for frailty screening. It was reported that frailty is strongly associated with cognitive impairment and clinically diagnosed dementia among persons aged 76 and older. It is possible that cognitive impairment is a clinical feature of frailty (Kulmala et al., 2014).

### Institutionalization

The possibility of dementia should be considered in individuals who do not make a call by looking up phone numbers, cannot manage their own deposits and savings at the bank or ATM, and repeatedly say the same things. Our findings suggest that these individuals should consider visiting a physician to rule out the possibility of dementia or receive early intervention if found to have dementia.

### Strength and limitations

Strengths: this study was performed with a relatively large sample size of 1,873 individuals. Our findings suggest that individuals who do not make a call by looking up phone numbers, cannot manage their own deposits and savings at the bank or ATM, cannot tell what the day, and repeatedly say the same things are important early signs of dementia.

## Limitations

First, although many patients tested positive on the screening test and were advised to see a doctor, a small percentage visited a doctor. However, a logistic analysis could have helped overcome this limitation; moreover, the number of patients who underwent the MMSE was sufficient for analysis. Second, the exact diagnoses of our participants were unclear since we could not obtain radiological findings, including magnetic resonance imaging (MRI) findings. Third, we could not obtain information regarding the educational status, and the patients were not assessed using the Geriatric depression scale. Fourth, we could not obtain information regarding whether the participants live with their family or live alone. If they live alone, they have to manage their own deposits and savings at the bank or ATM. However, if they live with their family, their family may not allow them to draw the deposit by themselves. In Japan, family members managing the deposits of elderly people have also been the cause of fraudulent withdrawals and spending of deposits without their permission. Finally, the individuals did not undergo blood tests, including tests for thyroid function and vitamin B levels (Hama et al., 2020; Ueno et al., 2022).

## Conclusions

The possibility of dementia should be considered in individuals who do not make a call by looking up phone numbers, cannot manage their own deposits and savings at the bank or ATM, and cannot tell what day today.

## Data Availability Statement

The raw data supporting the conclusions of this article will be made available by the authors, without undue reservation.

## Ethics Statement

The studies involving human participants were reviewed and approved by Institutional Review Board of the University of Fukui (No. 1007, February 27, 2014). The patients/participants provided their written informed consent to participate in this study.

## Author Contributions

TH carried out the studies, participated in collecting data, and drafted the manuscript. TH, MN, RM, YI, TI, and AI selected the self-reporting yes/no survey of dementia screening. YF and YN amended the manuscript. YK and TT performed the statistical analysis. SE, YE, AU, NS, MI, and KH participated in collecting data. All authors contributed to the article and approved the submitted version.

## References

[B1] Arabi Z. Syed Abdul Rahman S. A. Hazmi H. Hamdin N. (2016). Reliability and construct validity of the early dementia questionnaire (EDQ). BMC Geriatr. 16:202. 10.1186/s12877-016-0384-1 27903242 PMC5131430

[B2] Borson S. Scanlan J. Hummel J. Gibbs K. Lessig M. Zuhr E. (2007). Implementing routine cognitive screening of older adults in primary care: process and impact on physician behavior. J. Gen. Intern. Med. 22, 811–817. 10.1007/s11606-007-0202-8 17447100 PMC2219855

[B3] Boustani M. Callahan C. M. Unverzagt F. W. Austrom M. G. Perkins A. J. Fultz B. A.. (2005). Implementing a screening and diagnosis program for dementia in primary care. J. Gen. Intern. Med. 20, 572–577. 10.1111/j.1525-1497.2005.0126.x 16050849 PMC1490164

[B4] Campbell J. C. Ikegami N. (2000). Long-term care insurance comes to Japan. Health Aff. (Millwood) 19, 26–39. 10.1377/hlthaff.19.3.26 10812779

[B5] Chambers L. W. Sivananthan S. Brayne C. (2017). Is dementia screening of apparently healthy individuals justified? Adv. Prev. Med. 2017:9708413. 10.1155/2017/9708413 28932605 PMC5591898

[B6] Dunn B. Stein P. Cavazzoni P. (2021). Approval of aducanumab for Alzheimer disease-the FDA’s perspective. JAMA Intern. Med. 181, 1276–1278. 10.1001/jamainternmed.2021.4607 34254984

[B7] Durant J. Leger G. C. Banks S. J. Miller J. B. (2016). Relationship between the activities of daily living questionnaire and the montreal cognitive assessment. Alzheimers Dement. (Amst) 4, 43–46. 10.1016/j.dadm.2016.06.001 27489879 PMC4961826

[B9] Eichler T. Thyrian J. R. Hertel J. Wucherer D. Michalowsky B. Reiner K.. (2015a). Subjective memory impairment: no suitable criteria for case-finding of dementia in primary care. Alzheimers Dement. (Amst) 1, 179–186. 10.1016/j.dadm.2015.02.004 27239503 PMC4876911

[B8] Eichler T. Thyrian J. R. Hertel J. Michalowsky B. Wucherer D. Dreier A.. (2015b). Rates of formal diagnosis of dementia in primary care: the effect of screening. Alzheimers Dement. (Amst) 1, 87–93. 10.1016/j.dadm.2014.11.007 27239495 PMC4876881

[B300] Folstein M. F. Folstein S. E. McHugh P. R. (1975). Mini-mental state. A practical method for grading the cognitive state of patients for the clinician. J. Psychiatr. Res. 12, 189–198. 10.1016/0022-3956(75)90026-6 1202204

[B10] Galvin J. E. (2015). The quick dementia rating system (QDRS): a rapid dementia staging tool. Alzheimers Dement. (Amst) 1, 249–259. 10.1016/j.dadm.2015.03.003 26140284 PMC4484882

[B11] GBD 2019 Dementia Forecasting Collaborators. (2022). Estimation of the global prevalence of dementia in 2019 and forecasted prevalence in 2050: an analysis for the global burden of disease study 2019. Lancet Public Health 7, e105–e125. 10.1016/S2468-2667(21)00249-8 34998485 PMC8810394

[B12] Hama Y. Hamano T. Shirafuji N. Hayashi K. Ueno A. Enomoto S.. (2020). Influences of folate supplementation on homocysteine and cognition in patients with folate deficiency and cognitive impairment. Nutrients 12:3138. 10.3390/nu12103138 33066591 PMC7602498

[B13] Harrison J. K. Fearon P. Noel-Storr A. H. McShane R. Stott D. J. Quinn T. J. (2014). Informant questionnaire on cognitive decline in the elderly (IQCODE) for the diagnosis of dementia within a general practice (primary care) setting. Cochrane Database Syst. Rev. 7:CD010771. 10.1002/14651858.CD010771.pub2 24990271

[B14] Ideno Y. Takayama M. Hayashi K. Takagi H. Sugai Y. (2012). Evaluation of a Japanese version of the mini-mental state examination in elderly persons. Geriatr. Gerontol. Int. 12, 310–316. 10.1111/j.1447-0594.2011.00772.x 22122408

[B15] Johnson N. Barion A. Rademaker A. Rehkemper G. Weintraub S. (2004). The activities of daily living questionnaire: a validation study in patients with dementia. Alzheimer Dis. Assoc. Disord. 18, 223–230. 15592135

[B16] Kitamura M. Izawa K. P. Ishihara K. Brubaker P. H. Matsuda H. Okamura S.. (2022). Differences in health-related quality of life in older people with and without sarcopenia covered by long-term care insurance. Eur. J. Investig. Health Psychol. Educ. 12, 536–548. 10.3390/ejihpe12060040 35735461 PMC9222037

[B17] Kulmala J. Nykänen I. Mänty M. Hartikainen S. (2014). Association between frailty and dementia: a population-based study. Gerontology 60, 16–21. 10.1159/000353859 23970189

[B18] Lang L. Clifford A. Wei L. Zhang D. Leung D. Augustine G.. (2017). Prevalence and determinants of undetected dementia in the community: a systematic literature review and a meta-analysis. BMJ Open 7:e011146. 10.1136/bmjopen-2016-011146 28159845 PMC5293981

[B19] Le Couteur D. G. Doust J. Creasey H. Brayne C. (2013). Political drive to screen for pre-dementia: not evidence based and ignores the harms of diagnosis. BMJ 347:f5125. 10.1136/bmj.f5125 24018000

[B20] Ohara T. Hata J. Yoshida D. Mukai N. Nagata M. Iwaki T.. (2017). Trends in dementia prevalence, incidence and survival rate in a Japanese community. Neurology 88, 1925–1932. 10.1212/WNL.0000000000003932 28424272

[B21] Prince M. Bryce R. Albanese E. Wimo A. Ribeiro W. Ferri C. P. (2013). The global prevalence of dementia: a systematic review and meta analysis. Alzheimers Dement. 9, 63–75.e2. 10.1016/j.jalz.2012.11.007 23305823

[B22] Satake S. Senda K. Hong Y.-J. Miura H. Endo H. Sakurai T.. (2016). Validity of the Kihon checklist for assessing frailty status. Geriatr. Gerontol. Int. 16, 709–715. 10.1111/ggi.12543 26171645

[B23] Thyrian J. R. Eichler T. Pooch A. Albuerne K. Dreier A. Michalowsky B.. (2016). Systematic, early identification of dementia and dementia care management are highly appreciated by general physicians in primary care-results within a cluster-randomized-controlled trial (DelpHi). J. Multidiscip. Healthc. 9, 183–190. 10.2147/JMDH.S96055 27143912 PMC4844257

[B24] Thyrian J. R. Fiß T. Dreier A. Böwing G. Angelow A. Lueke S.. (2012). Life- and person-centred help in mecklenburg-western pomerania, Germany (DelpHi): study protocol for a randomised controlled trial. Trials 13:56. 10.1186/1745-6215-13-56 22575023 PMC3482148

[B25] Ueno A. Hamano T. Enomoto S. Shirafuji N. Nagata M. Kimura H.. (2022). Influences of vitamin B_12_ supplementation on cognition and homocysteine in patients with vitamin B_12_ deficiency and cognitive impairment. Nutrients 14:1494. 10.3390/nu14071494 35406106 PMC9002374

[B26] Woods B. Aguirre E. Spector A. E. Orrell M. (2012). Cognitive stimulation to improve cognitive functioning in individuals with dementia. Cochrane Database Syst. Rev. 13:CD005562. 10.1002/14651858.CD005562.pub2 22336813

